# The Physiology of Reproduction – *Quo vadis*?

**DOI:** 10.3389/fphys.2021.650550

**Published:** 2021-03-30

**Authors:** Richard Ivell, Ravinder Anand-Ivell

**Affiliations:** School of Biosciences, University of Nottingham, Nottingham, United Kingdom

**Keywords:** hormonal networks, parameter variance, pulsatility, endocrine disruptors, uterine peristalsis, systems biology

## Abstract

The reproductive system in males and females reflects a highly dynamic underlying physiology. Yet our current understanding of this system is still largely based upon relatively simplistic snapshots of individual component cells and tissues. Gamete production as well as gonadal hormone synthesis and its influence are the manifestations of dynamic and redundant informational networks and processes, whose qualitative and quantitative dimensions, especially through development from embryo through puberty and adulthood into ageing, are still largely uncharted. Whilst the recent huge advances in molecular science have helped to describe the components of the reproductive system in ever greater detail, how these interact and function in space and time dimensions is still largely obscure. Recent developments in microfluidics, stem cell biology, and the integration of single-cell transcriptomics with tissue dynamics are offering possible methodological solutions to this issue. Such knowledge is essential if we are to understand not only the normal healthy functioning of this system, but also how and why it is affected in disease or by external impacts such as those from environmental endocrine disruptors, or in ageing. Moreover, operating within a complex network of other physiological systems, its integrational capacity is much more than the generation of male and female gametes and their roles in fertility and infertility; rather, it represents the underpinning support for health and well-being across the lifespan, through pregnancy, puberty, and adulthood, into old age.

## Introduction

Physiology has been defined as the “*science of health*” as well as the “*science of life*.” For reproductive physiology, this is not unproblematic since much of what we consider as reproduction is not essential to either. At an individual level, disorders of the reproductive system such as infertility are generally not life-threatening, nor lead to major bodily dysfunction. Whilst some might consider that infertility is not a disease, at the population level, any disruption of reproductive function can lead to loss of a population or even a species. And at the individual level, such malfunction can lead to considerable personal grief. Accordingly, in the competitive jungle of research funding, reproduction does not compete well with cancer, diabetes, or neurodegenerative illness, even though fertility problems may impact a larger number of people, especially as child-bearing is being delayed until the mid to late 30s or beyond. Our field is further disadvantaged by a minimal engagement from the pharmaceutical industry, for whom the reproductive sector is a litigious minefield because of the subtle risks associated with exposures of an unborn child. Consequently, most drugs used today still have their roots in substances discovered many decades ago, and which if invented today would probably find it difficult and expensive to receive approval. Finally, the relative success of Assisted Reproductive Technology (ART) has lulled society into thinking that infertility issues are largely resolved.

A consequence of such attitudes is that research into fundamental reproductive physiology has not progressed as much as in other fields and owes its advances often to translation from different sectors of physiology. Moreover, when we assess the articles being submitted to our specialist *Frontiers* section on “Reproduction,” by far the majority represent incremental improvements to existing clinical procedures and applications, and relatively few contributions to innovative basic science.

In this essay, we should like to draw attention to why Reproductive Physiology merits more fundamental research – in particular, into those aspects which differ from other areas of Physiology. This is intended neither as a comprehensive review of recent breakthroughs and successes, nor as a detailed list of future research targets. Rather, we want to raise ideas to stimulate innovative approaches to understanding the field as well as its applications into a broad array of translational targets.

## The Dynamic Physiology

Most areas of physiology are governed by a relatively static image of an adult system, whose rules and boundaries are controlled by homeostasis. There is a normal (healthy) state where components have fixed relationships to one another, which may become altered in a disease condition. In contrast, the reproductive system is never static. In the adult female, reproductive status is ruled by the dynamic depletion of the follicle reserve, from birth to old age, superimposed upon which is the menstrual cycle, or its equivalent, regulating not only the final preparation of the egg for fertilisation and eventual pregnancy, but also the organs of the reproductive system such as the uterus and mammary glands, as well as other systems in the body such as the bone and skeletal system, the osmotic balance, the immune system, and especially the brain and behaviour. In the male, there may be no monthly cyclicity, nor limited gamete reserve, but the system is still very dynamic, with continued spermatogenesis from stem cells throughout adult life. With the possible exceptions of intestinal epithelial sloughing and haematopoiesis, no other organ system is adapted to producing continually many millions of cells a day whose function is external to the organism, and which must be capable of generating perfectly functional individual cells (spermatozoa) for many decades.

The parameters governing both male and female reproductive systems – even in the adult – are therefore never static, but to be seen always as snapshots of very dynamic processes, the dimensions of which can at best only be approximated, much as a photograph can never display the living reality of the subjects.

However, the reproductive system also reciprocally dictates the health of the organism, not only in adulthood, but especially during prenatal development, puberty, and in senescence, adding further dimensions to the dynamic interplay of the cells and organs involved. Events occurring early in development can have big effects at later stages, as in the DOHaD (Developmental Origins of Health and Disease) concept. It is our lack of understanding of the dynamic processes involved and their regulation which may account for the fact that many of the commonest diseases affecting us are those involving the reproductive system and its dynamic development (polycystic ovarian syndrome, endometriosis, fibroids, premature ovarian insufficiency, male and female infertility, breast, endometrial, and ovarian cancers, benign prostatic hyperplasia, testis and prostate cancers, and hypoandrogenemia).

An aspect of this, which is not often appreciated, is the large variation in clinical and biological parameters in the human population associated with reproduction, which we consider “normal” and non-pathological. Part of this may reflect the point raised above that, since disruption of the reproductive system is rarely life-threatening, we are more tolerant of variation *per se*. For the male, for example, the WHO latest acceptable reference range for sperm concentration is 15–259 million per ml with progressive motility of 32–75% and for normal morphology is only > 4% (Cooper et al., 2010; WHO, 2010)! We have shown that the normal range for circulating insulin-like peptide 3 (INSL3), a measure of Leydig cell functional capacity (i.e., total number of Leydig cells in the testes compounded with their differentiation status) is 0.39–1.87 ng/mL (5%, 95% confidence intervals) in healthy young men (Anand-Ivell et al., 2021). This is a large variation, compared to other, comparable non-episodic health parameters, such as thyroxine (5.5–12.5 μg/dL) or haemoglobin (14.0–17.5 g/dL). For women, there is a similar large range for the age at natural menopause (40–58 years) or circulating AMH (6.4–67.9 pmol/L in reproductive age women), both a reflection of egg reserve. It is important to note that these are quasi-constitutive parameters not subject to acute feedback control, for example, by the hormones of the hypothalamo-pituitary-gonadal (HPG) axis. Yet we understand very little about the causative factors involved in this large variation in reproductive parameters, except that they probably reflect events early in development either before birth or during puberty. Nor do we fully understand the roles played by ethnicity and genetics in establishing these normal ranges.

Such reproductive parameters probably depend upon stem cell proliferation and differentiation, the important features being the relative timing of these two processes and how that timing is controlled. There are similarly dynamic mechanisms affecting cell death (apoptosis) in these lineages. Thus, reproductive physiology must be seen as a complex interaction between multiple highly dynamic processes. Yet most of our knowledge and understanding is based upon sporadic snapshots of mostly adult scenarios.

A further exacerbating factor is that we know most about very few species, such as the human or the mouse, and then try to extrapolate and adapt this knowledge to other animals. Whilst this may be reasonable for some species, such as ruminants, or primates, it can become problematic for exotic and endangered species, or lower vertebrates, whose physiology is often nebulous at best. There is therefore a need for elaborating more and alternative animal models to help delineate these complex processes.

## Information Theory 101

Information theory, originally derived from telecommunication to describe information transfer and its efficiency, is highly pertinent to any understanding of how living systems work, yet has surprisingly found little mention in basic biology texts. The flow of information – its production and its reception – is the key difference between living and non-living, between life and death, and between health and disease (Yockey, 1992). Because the reproductive system is, by definition, very ancient and essential for the survival of the species, we see a wide range of informational systems being used, both paracrine and endocrine. The HPG axis represents a classic example for information transfer at a distance. Not only are the gonadotropin hormones that are produced by the pituitary gland, complex information carriers whereby multiple peptide and carbohydrate epitopes within the same molecule all need to be correctly “seen” by their cognate receptors, thus minimising misinformation, but the system additionally employs both pulsatility (digital encoding) and negative feedback to ensure correct and adequate information transfer.

Both gonadal steroids and certain peptide hormones (e.g., inhibin B) feed back to the hypothalamus and pituitary to regulate the output signal. Whilst we now understand much of the qualitative and anatomical detail, surprisingly, we know only very little about the quantitative aspects involved here. How do those cells receiving the feedback quantitatively measure the amounts of gonadal steroids, inhibin B, or perhaps other molecules feeding back information; how are these feedback signals quantitatively integrated to generate not just a yes/no secretion response but an appropriate quantity of a particular hormone by the hypothalamus and/or pituitary? Regarding pulsatility, we still know very little about the detailed mechanisms involved. It is 40 years since the pioneering study by Wildt et al. (1981) which showed for macaques that GnRH pulse frequency determined the quantities of gonadotropins released and whether preferentially FSH or LH or both. Although Johannes Veldhuis and colleagues have elaborated examples of the changes that occur in gonadotropin pulsatility with development, sickness, or ageing (e.g., Veldhuis et al., 1994; Liu et al., 2005), little of this knowledge has yet been practically translated into clinical application. We still rely upon single measurements of peripheral hormones, hopefully at a similar time of day, or at best a GnRH challenge test. There is obviously a major technical obstacle here: how can we measure and interpret pulsatility in a routine clinical context? Only few studies have tried to approach this issue (e.g., Henley et al., 2009; Liang et al., 2019; Poudineh et al., 2020). Given our advances in other areas of technology, it is surprising that we seem to be no nearer to harnessing the informational content in pulsatility, especially when we consider how many different hormone systems appear to make use of it and how important it might be for the understanding of health and disease. We could learn from findings from other pulsatile hormones, such as parathyroid hormone, where loss of appropriate pulsatility (rather than its concentration) is a biomarker of osteoporosis (Chiavistelli et al., 2015). Conversely, administration of hormones or drugs in a pulsatile fashion is still very limited. Rather, hormones, such as GnRH analogues, may be administered as a single, high-concentration bolus specifically and chronically to desensitise and hence inactivate their receptors and the relevant feedback loop. Even for oxytocin, another well-characterised pulsatile hormone, it is still advocated at labour to deliver it as a continuous rather than a pulsed infusion (e.g., Tribe et al., 2012); even though it requires higher amounts of the hormone and can lead to oxytocin receptor desensitisation, including possibly within the foetal brain and leading to a risk of autism (e.g., Weisman et al., 2015). A greater understanding of how hormones are encoded and decoded in pulsatile systems could open up many new opportunities for drug-based therapies, besides expanding our understanding of disease.

## Networks, Noise, and Variance

Historically, the great successes achieved in combatting disease with single drug replacement strategies, such as the application of insulin in diabetes, or thyroxine in hypothyroidism, have created a dogma that most physiological systems can be similarly “fixed” by activation of a simple pathway; conversely, that if a gene product is important, its knockout or removal must show a clear phenotype. The reality that is especially being highlighted by the “big data” approaches of GWAS studies, or global NGS-sequencing, is that many genes may be involved in a particular phenotypic pathway, none of which individually may deserve the appellation of being “causal” or “essential.” Rather genes and their products are linked together into complex and dynamic networks whose functionality can tolerate a degree of redundancy, but which are nevertheless holistically essential for the appropriate function of a cell or tissue.

A good example is provided by the dialogue between the blastocyst and the endometrial lining of the uterus at the time of implantation. We now understand that mutual and multiple signals from both the blastocyst as well as from the receptive endometrium interact in a largely temporally sequential fashion, subsequent steps being “mostly” dependent on successful “hand-shaking” of preceding steps. The term “mostly” is highlighted because this system allows a modest redundancy, both in molecular and in temporal terms. Only when the negotiation is satisfactorily finalised can the blastocyst be considered as implanted and placentation can proceed. The negotiation involves quality checks: on the embryo to determine whether it has the potential to develop into a healthy offspring and on the mother to test whether she will be able to take this baby to term. If the quality checks fail, then there is miscarriage, preferring to reserve energy for a new pregnancy rather than risking failure at a later stage (e.g., Macklon and Brosens, 2014; Valdes et al., 2017; Craciunas et al., 2019; Ewington et al., 2019).

In such a system, it is difficult to identify single key molecules or biomarkers essential to successful early pregnancy; in general, the dynamics of the negotiation process are very hard to measure. This is even more difficult in species where the apposition time of the embryo to the uterine lining is much longer than in the human. In analogy to the “quiet embryo” hypothesis (Baumann et al., 2007), it is likely that when the negotiation is rapid and signalling within this network is relatively quiet, it is a sign of a healthy implantation process; in contrast when it is protracted or “noisy” with much information flow, this could be an indication that the process is problematic with a high risk of failure. But how can we assess such a dynamic process? Importantly, how can we manipulate this process, and should we? By overriding this quality control dialogue, there is a risk that we might be retaining an offspring which otherwise might have been subject to natural selection. But what if the quality control process itself is faulty? One consequence of this hypothesis might be that molecular parameters will show a higher level of “noise,” or variance, with a problem outcome, though may not show alterations in mean parameter levels.

Consider, for example, the situation during ART when multiple healthy-looking embryos are transferred and only one succeeds to implant. Was the decision here a quantitative one, or qualitative, or a dynamic combination of these?

A related example concerns the extent to which informational processes may involve stochastic mechanisms. During the early cell divisions of the fertilised egg, the resulting morula comprises more-or-less identical blastomeres, each of which can, if separated, give rise to an independent individual. Early blastomeres are totipotent. There are few geographical clues until the blastocyst stage, except for those resulting from the point of sperm entry, and any unequal distribution of the original egg cytoplasm and its contained gene products. Only later, with the formation of the blastocoel, inner and outer layers, and the initial trophoblast, can clear polarity be established, and cell lineages become determined. However, attempts to use individual blastomeres as diagnostic tools to deliver prognosis on the resulting embryo have generally not succeeded, largely due to the very heterogeneous nature of blastomere gene expression profiles. One explanation for this could be that initially gene expression in the early morula is mostly a stochastic process rather than being deterministic. The blastomere genomes are mostly not epigenetically determined once the zygote is formed and will anyway undergo an erasure phase in the blastocyst. The stochastic activation of genes would have two consequences: first, it could establish a random informational (chaos) network which could then progressively by feedback fixate upon certain pathways and relationships and second, it could provide an active mechanism for proof-reading the genome. Both mechanisms would have definite advantages for the survival of the offspring. From a pragmatic perspective, such a stochastic mechanism would also explain why there may be an upper limit to the success rate of *in vitro* fertilisation (IVF), since any such proof-reading step implies the necessary loss of inadequate zygotes. But such a mechanism would also explain why IVF succeeded in the first place, since it would intrinsically be able to rectify any potential damage caused by the unusual IVF environment. It will be very interesting to find a way to test such a paradigm and the resulting informational networks and thus redefine the impact of *in vivo* and *in vitro* culture conditions, especially in the context of the infertile patient or the endangered species.

## Development: Blurring the Boundaries

The relatively recent relaxation of socio-political boundaries has introduced the concept of gender fluidity. Until only a few years ago, scientists were seeking and finding molecular mechanisms which by mutual inhibition would ensure the polarity of the sexes and thus the prerequisite for reproduction and the continuation of the species. Classic examples of this are the expression of the SRY gene on the Y chromosome (Koopman et al., 1990), or the reciprocal inhibition of FGF9 and Wnt4 (Kim and Capel, 2006) during early gonad development. The demonstration of such examples unconsciously suggests that subsequent unclear sex is likely due to aberrations of development, with research then focussed on singular defects of an otherwise perfect system. However, we now realise that gender physiology is not as polarised as we once imagined; it appears more plastic, whereby dynamic and complex informational networks, as discussed above, can be influenced in more than one direction. This would allow a degree of redundancy in the system and a possible blurring of the boundaries.

To illustrate the issue let us consider the foetal gonadal hormones. We are used to considering that phenotypic androgenisation is brought about by testosterone produced by the foetal testes within the so-called “masculinisation window” (Welsh et al., 2014). Less well known is the fact that female foetuses produce almost half the concentration of testosterone as male foetuses (Ivell et al., 2017), mostly from the foetal adrenal gland, and certainly more than sufficient to influence androgen-sensitive systems. Not only that, but in multiparous species such as rodents or pigs, gonadal hormones like testosterone or the peptide INSL3 can transfer between male and female foetuses *in utero* (Vernunft et al., 2016), without having any apparent effect on the female phenotype. It is argued that in the female foetus, the receptors for these hormones are suppressed and therefore unresponsive, but then we need to ask why the androgen receptor in mammals has been evolutionarily segregated to the X chromosome, where there is a greater likelihood of it being expressed in females or recessively mutated in males. Could the solution be that whilst we know a lot about the qualitative response of cells to hormones, we still know very little about the quantitative response: how much hormone is sufficient to elicit an appropriate response *in vivo*? Evidently, we still understand far too little about the qualitative and quantitative complexity of the informational networks governing the phenotypic expression of gender.

We also need to consider why the reproductive system has conserved the components of the HPG axis across the whole of chordate evolution, and yet when it comes to sex determination appears to employ a wide diversity of genetic systems in mammals, birds, and reptiles. For a physiological system which is absolutely essential for the survival of the species, this is indeed surprising, and suggests that there is much that we still do not comprehend.

## Endocrine Disruptors

Reduced reproductive performance was one of the first physiological systems to be recognised as being negatively influenced by endocrine disrupting chemicals (EDCs) in the environment (Gore et al., 2015). These pervasive manmade substances (pesticides, plasticisers, fire retardants, microplastics, etc.) are now known to influence most organ systems of the body, often at very low exposures and concentrations, and ambient levels are increasing. Importantly, they appear not to conform to conventional toxicological paradigms, but can show non-monotonic dose–responsiveness even down to very low concentration. Moreover, because of epigenetic changes, they may also impact later life periods and subsequent generations. Among their key characteristics (La Merrill et al., 2020), they appear to function by interfering with diverse endocrine components (hormones, receptors, transporters, enzymes, etc.). However, in the light of what has been discussed earlier, they may function additionally by subtly modulating informational networks. One consequence of this is that effects may be due less to absolute changes in endocrine parameters and more to increased noise or variance in such informational systems. Such an effect was suggested in assessing the effect of phthalate exposure on the risk of cryptorchidism or hypospadias in male human foetuses (Anand-Ivell et al., 2018).

The biggest problem to be faced with regard to EDCs is that they often appear to be insensitive to conventional regulation applying toxicological paradigms. Regulatory agencies require more and novel approaches if they are to be able to keep up with the many millions of tons of such substances released into the environment every year. While some *in vitro* or *in silico* methods are showing considerable promise, these work only by assuming that EDCs are mimicking natural hormones and their interactions with receptors. Yet, as we have shown above, EDCs appear to illustrate a range of diverse endocrine properties, and moreover may function within the context of dynamic and redundant networks, so that such relatively simplistic approaches may fail to recognise a majority of potential EDC compounds.

A further complication in the interaction between EDCs and reproductive physiology is that we now recognise that also many pharmaceutical compounds are behaving similarly. Initially, it was discovered that the contraceptive component ethinyl oestradiol was entering natural water systems relatively unscathed and having potent effects to feminise male fish (Jobling et al., 2006). Therefore, precisely those properties which make a pharmaceutical resistant to degradation in the body are also those that allow it to persist in the environment once excreted (Heberer, 2002). Thus, our marine and freshwater environments are having to cope with increasing amounts of common pharmaceuticals, including contraceptive agents, analgesics, hypertensive drugs, etc. Their impact on food chains is still largely uncharted.

Also of concern is that we are discovering that many pharmaceuticals, previously considered relatively harmless, are now seen as impacting on foetal development. The analgesics, paracetamol (acetaminophen) and ibuprofen, have been shown in animal models as well as in human epidemiological studies to affect the development of the foetal gonads following exposures during pregnancy (Kristensen et al., 2016; Kilcoyne and Mitchell, 2019). The issue here is that previous regulatory strictures regarding testing evidently did not take account of the subtle mode of action of EDCs, particularly on the reproductive system or during pregnancy. Research is still at a very early stage here, and it is likely that the future will reveal that several common or over-the-counter pharmaceuticals may be found to have EDC-like effects. Particularly concerning in this regard is also the massively increased prescription of psychoactive drugs, such as SSRIs, to women of reproductive age, and to adolescents going through puberty (Fenger-Grøn et al., 2011; Grzeskowiak et al., 2012). These are categories which we know are likely to be very sensitive to the effects of potential endocrine-acting compounds.

## Age and Reproduction

It is the purpose of any species, once it has reached sexual maturity, to reproduce and secure the continuation of the gene pool. Humans are different. The attainment of sexual maturity at the end of puberty is separated by several years both from parenthood itself, as well as from the behavioural maturation that is required for a successful family. There are three sequelae to this. First, women experience a succession of futile physiological preparations for pregnancy without ever becoming pregnant. Evolution has not prepared women for this. Every month cyclic hormones activate stem cells to begin preparing the endometrium or breast tissue for an eventual pregnancy without this event actually occurring, and a default programme is then invoked to reset the calendar. Besides the accompanying menstruation and/or mental disruption this provokes, it has been also suggested that this activation/reset system is one factor predisposing women to reproductive tissue cancer (Olsson and Olsson, 2020). Second, contraceptive systems have been developed to provide planning for reproduction to coincide with an optimal economic environment. The commonest contraceptives for women still rely on manipulating the cyclic hormones following a paradigm which is now more than 60 years old. Whilst choice and alternatives also for men are needed, few new concepts have found acceptance. Third, socio-economic pressures have pushed the age at which families are started into the mid-30s or older. Fertility in women declines rapidly from the early 20s onwards, with mean TTP (time-to-pregnancy with unprotected intercourse) shifting from about 3 months at age 20–25, to 6 months or longer at 30–35 years, and >24 months by the age of 41. Whilst modern ART procedures have done much to alleviate such infertility issues, and even the freezing of youthful ovarian tissue or oocytes has progressed to mainstream, there are inevitably consequences of such alterations of biology for which evolution has not prepared us. Maternal and paternal age is known to be not only associated with increased genetic risk for the offspring, but also subtle epigenetic effects which may only become apparent later in life (Aiken and Ozanne, 2014; Preston et al., 2018).

There is still much we do not understand about the physiological impacts of this shift in family planning. But increased life expectancy has also thrown emphasis on the endocrine roles played by the gonads in older age. The gonads and reproductive system not only serve the production of offspring, but their hormones contribute majorly to health and overall well-being. The exhaustion of the ovarian follicle reserve in women at mean age 51 years signals the menopause, though this is not an abrupt transition but may progress over several years as the resident follicles slowly lose their capacity to produce hormones. Until recently, loss of follicles was considered synonymous with loss of the sex steroids oestradiol and progesterone, and to date hormone replacement therapy (HRT) has focussed only on these two molecules. However, it is becoming clear that the follicles also produce other hormones, such as the gonadal peptides INSL3 or relaxin, which are also lost to the circulation at the menopause. Recent studies in mice and humans have shown that loss of INSL3 expression or of its specific receptor leads to osteopenia or osteoporosis (Ferlin et al., 2008), and relaxin has markedly positive effects on cardiovascular physiology and fibrosis (Leo et al., 2019), indicating that future HRT concepts might need to consider replacing additional hormones.

In men, similarly, the gonads also produce peptide hormones, such as INSL3, in addition to steroids like testosterone. Importantly, these also decline significantly with increasing age. Whereas testosterone declines at between 5 and 7% per decade, INSL3 declines at almost 15% per decade, since the latter, being quasi-constitutive, is not acutely compensated by increasing gonadotropins (Anand-Ivell et al., 2006) and is a true reflection of the age-dependent decline in Leydig cell functional capacity. Besides a possible role for INSL3 in osteoporosis in men, this decline in Leydig cell function adds to the growing debate about whether or not to supplement older men with testosterone. At present, HRT for men is mostly restricted to those exhibiting specific late-onset hypoandrogenemia (LOH), which affects only about 2% of elderly men and is characterised by specific androgen-deficiency symptoms (Basaria, 2013; Pye et al., 2014). Further research is evidently required to explore this age-dependent endocrine decline in men which accompanies increased frailty, cognitive decline, and increased incidence of metabolic syndrome. A new kind of HRT concept for men, with broad application, could go a long way to alleviating many of the debilitating ailments affecting men as they get older. In both men and women, the gonads are not just meant to produce gametes, but contribute to overall well-being and general health.

## Big Data

Recent years have witnessed a revolution in our ability to gain information about genes and their expression, with technologies such as GWAS analysis, MS-proteomics, NGS RNA-sequencing, as well as microarrays, allowing for the first time a global and comprehensive approach to measuring molecular events in cells, tissues, and organisms. In turn, this has led to many new statistical methods to distil out key events and pathways from these large datasets. Whilst no doubt providing much new information about physiology, these approaches still only achieve a molecular snapshot of what is going on. As alluded to the above, part of the difficulty is that we are still treating the objects of our study using simplistic paradigms and hypotheses, which ignore the dynamic nature, the complexity, and the redundancy, of what is driving physiology, particularly in reproductive tissues and organs. Many recent “big data” studies have not succeeded in, for example, identifying key biomarker(s) for healthy endometrium, or healthy follicles, or fertile sperm. Rather they illustrate redundancy, or place a probability on a pathway, with few if any studies coming up with equivalent and comparable results for a particular target. The problem is not the methodology, but the over-simplistic hypotheses that we apply. Evolution has had many millions of years to perfect informational systems which are robust, redundant, highly dynamic, and flexible. Such systems are rarely binary, often subtle, reflecting small changes in sensitivity, and can involve shifts in synchrony as much as quantitative shifts of expression. We need to invest research into a better understanding of such systems, using computer modelling and systems approaches in addition to discovery in order to extend our frontiers.

## Physiology Is More Than Molecules, E.G., Uterine Peristalsis

Molecular analysis has dominated recent decades of research in reproduction and has the advantage of providing hard, quantifiable data. It maps the molecular skeleton upon which physiological forces can act. But it does not answer the question of how things work. This deficit may explain in part why reproduction as a clinical discipline is still reliant on many surgical procedures as treatments, such as hysterectomy, prostatectomy, or caesarean section.

A good example is provided by the topic of uterine peristalsis. Like the gut, the uterus is also enclosed within sequential layers of longitudinal and circular smooth muscle and is subject to directed peristaltic waves of contraction. At the most receptive time of the menstrual or oestrous cycle, these contractions are mostly in the direction cervix to fundus (i.e., from bottom to top) coinciding with the opening of the valve or isthmus between the uterus and the oviduct on the ovarian side producing a fertile oocyte. This is the optimal time for intercourse, and a bolus of ejaculated sperm will pass the cervix and be transported by a peristaltic wave to the open isthmus and to the oviduct for fertilisation (Kunz and Leyendecker, 2002; Kuijsters et al., 2017). Pituitary oxytocin and uterine oxytocin receptors may also be involved in this contractility (Kunz et al., 2007). Later in the cycle, the directionality of uterine peristalsis reverses; the isthmus to the oviduct is closed and contractions serve to flush out menstrual fluid through the cervix. It is believed that the condition, endometriosis, whereby endometrial tissue grows ectopically on peritoneal membranes or on the ovarian surface, may be initiated by a dysperistalsis causing menstrual debris including endometrial stem cells to be flushed in the wrong direction through the oviduct and into the peritoneal cavity (Child and Tan, 2001). Similarly, uterosalpingography, and more recently cine-MRI, have shown that in infertile women, instead of normal cervix-to-fundal peristalsis at ovulation, the waves are reversed or irregular, thereby preventing sperm from fertilising the egg. Moreover, it is believed that the presence of fibroids is also a key factor in disrupting uterine peristalsis (Pier and Bates, 2015). Furthermore, in some species, producing multiple embryos, regular, standing peristaltic waves may be responsible for spacing of the embryos within the uterus.

We know very little about how uterine peristalsis is controlled. In analogy to the gut, it appears that there may be resident Cajal-like cells, or telocytes, in the uterus, which acting as pacemakers could coordinate myometrial contractility, in conjunction with the autonomic nervous system (Hutchings et al., 2009; Banciu et al., 2016). There does not appear to be any steroid-induced geographic expression of oxytocin or other receptors. Considering how important the observations described above may be in relation to some of the major diseases affecting human reproduction, endometriosis, fibroids, and/or infertility, as well as conditions such as premature labour, it is perhaps surprising that so little is known about this classical aspect of physiology. But then this is not an aspect which can be easily assessed using modern molecular techniques. New developments in cine-MRI are making the assessment of uterine peristalsis more accessible, though this is still some way from understanding causality or identifying possible treatments.

At a cellular level, it has recently been shown for the rat uterus, by combining electrophysiology using microelectrode arrays and microanatomy, that contractility in late gestation appears to be initiated by a “pacemaker” domain anatomically defined as a specialist region of the myometrium adjacent to placental tissue (Lutton et al., 2018). How such domains function and whether the concept applies to other times besides late gestation, or in other species, remains to be clarified.

## Biodiversity, Behaviour, and the Bigger Picture

The loss of biodiversity and loss of endangered species are also aspects touching the physiology of reproduction. This may be direct in that the reproductive system of a particular species is suffering dysfunction through exposure to environmental chemicals, or genetic inbreeding and an environment to which the species is not well adapted. Or it may be indirect because the behavioural context for appropriate reproductive physiology is dysfunctional. For example, it has been shown that EDC exposure can upset mating behaviour in rats without having any other obvious molecular or physiological consequences. Without going into detail, it is important to note that only when an integrated physiological approach is taken, which considers all aspects pertaining to reproduction (genetics, behaviour, ecology, endocrinology, and reproductive physiology) will it be possible to understand and thus develop solutions to the current biodiversity problem. While modern approaches to develop ART procedures suitable for endangered species, such as rhinoceros, are essential and laudable, only when these can be integrated into the larger physiological and environmental context of habitat renewal and behavioural monitoring, can any success be expected. Thus, the importance of taking a physiological approach in reproductive science is that it – by definition – is integrative and inclusive of a wide range of methodologies, rather than being constricted by a discipline-based focus on the molecular or the genetic.

## Light on the Horizon—New Methods and Approaches

As outlined above, the static cell-molecule approach needs to transmute into a more dynamic and integrated systems methodology. The progression of stem cell biology and the development of lineage into more complex *in vitro* models using organoids are already providing important dynamic information about cell–cell interactions. Similarly, the evolution of microfluidics systems allowing informational integration between different organ components is beginning to illuminate the reciprocal dynamics of the oestrous cycle (Xiao et al., 2017). Combining multiple single-cell transcriptomics across tissue sections is highlighting the dynamic heterogeneity of the endometrium and the placental–maternal interface, besides for the first time functionally identifying the roles of individual cells in their natural context (Vento-Tormo et al., 2018; Garcia-Alonso et al., 2021). This new methodology, like the myometrial electrophysiology mentioned earlier, has in common that instead of homogenising tissue expression data, as in the past, it is celebrating the heterogeneity of single cells and their functionality, and combined with a systems biology computational approach, is allowing us for the first time to appreciate the spatial and temporal dynamics essential to reproductive function.

On a larger scale, mid-gestational foetal-maternal development has always represented a relatively inaccessible “black box,” whose disruption is considered critical in terms of maternal and foetal pathology (pre-term labour, preeclampsia, gestational diabetes, miscarriage, foetal organogenesis, and DOHaD). Access to placental, foetal, and amniotic samples for analysis, and also non-invasive methods are very limited. However, the recent breakthrough development of an artificial placenta and/or artificial womb is providing hope not only for the survival of ever-earlier pre-term infants, but also in terms of our understanding of the dynamic foetal–maternal interchange (De Bie et al., 2020).

## Concluding Remarks

This essay makes no attempt to be comprehensive or present a balanced summary of current research into reproductive science. Numerous excellent recent clinical and molecular advances have been ignored. Rather it attempts to show how our current focus on molecular detail may be obscuring our understanding of the mechanisms and processes which govern the functioning of our reproductive organs and tissues. These are highly dynamic and require a different kind of approach which is integrative in both space and time. Such new approaches will also probably require different statistical and computational methodologies, besides new techniques and new models. We have learnt to dissect and take apart the system into its component molecules, now it is time to put this knowledge back together again and understand how the complete system works.

## Author Contributions

RI and RA-I together conceived, authored, and reviewed the manuscript. Both authors contributed to the article and approved the submitted version.

## Conflict of Interest

The authors declare that the research was conducted in the absence of any commercial or financial relationships that could be construed as a potential conflict of interest.
